# Illness in Intensive Care Staff after Brief Exposure to Severe Acute Respiratory Syndrome

**DOI:** 10.3201/eid0910.030525

**Published:** 2003-10

**Authors:** Damon C. Scales, Karen Green, Adrienne K. Chan, Susan M. Poutanen, Donna Foster, Kylie Nowak, Janet M. Raboud, Refik Saskin, Stephen E. Lapinsky, Thomas E. Stewart

**Affiliations:** * Mount Sinai Hospital, Toronto, Ontario, Canada; † University Health Network, Toronto, Ontario, Canada

Severe acute respiratory syndrome (SARS) is a threat to healthcare workers. After a brief, unexpected exposure to a patient with SARS, 69 intensive-care staff at risk for SARS were interviewed to evaluate risk factors. SARS developed in seven healthcare workers a median of 5 days (range 3–8) after last exposure. SARS developed in 6 of 31 persons who entered the patient’s room, including 3 who were present in the room >4 hours. SARS occurred in three of five persons present during the endotracheal intubation, including one who wore gloves, gown, and N-95 mask. The syndrome also occurred in one person with no apparent direct exposure to the index patient. In most, but not all cases, developing SARS was associated with factors typical of droplet transmission. Providing appropriate quarantine and preventing illness in healthcare providers substantially affects delivery of health care.

Severe acute respiratory syndrome (SARS) is a disease that consists of fever and respiratory symptoms that can progress to respiratory failure and death (*1*). SARS is most likely to develop in healthcare workers and household or family contacts of infected persons (*2*–*4*) Unprotected exposure to SARS in hospitals has several potential consequences, which include the following: illness in persons and healthcare workers; transmission of SARS from ill healthcare workers and patients to visitors and household contacts; and reduced ability of the healthcare system to deliver care because of illness in or quarantine of healthcare workers. In addition, the psychological impact of isolation and quarantine can be substantial (*5*). As a result, understanding factors associated with SARS transmission after exposure to SARS patients is important and would assist with formulating appropriate quarantine procedures. We describe our experience with a large number of healthcare workers who were exposed to a patient in an intensive-care unit (ICU) with undiagnosed SARS.

## Index Patient

On March 23, 2003, a 74-year-old immunocompromised man was transferred to our ICU from a hospital where the original cluster of Toronto’s SARS cases occurred (*2*). The patient originally had signs and symptoms consistent with a presumptive diagnosis of community-acquired pneumonia. Before transfer, SARS was excluded from the differential diagnosis because the patient had not traveled, had never left the emergency department of the referring hospital, and had only had a single recent outpatient visit to an area of the original hospital in which SARS had not been identified. Upon arrival in our ICU, the patient was placed in precautions for methicillin-resistant *Staphylococcus aureus* (MRSA) pending admission screening results (*6*). Therapy with broad-spectrum antimicrobial drugs was initiated. Humidified high-flow oxygen was administered for the first 5 h, noninvasive positive pressure ventilation by oronasal mask for the next 18.25 h, and invasive mechanical ventilation for the subsequent time (7.5 h). Endotracheal intubation required fiber-optic placement. That the extent of the outbreak at the referring institution was larger than originally appreciated became apparent at this time; therefore, the patient was transferred to another facility for placement in negative pressure isolation for possible exposure to SARS. Subsequently, his family members became ill, and the SARS-associated coronavirus was identified in the patient’s respiratory secretions (polymerase chain reaction testing of bronchoalveolar lavage confirmed the diagnosis of SARS).

## Quarantine

Once the risk for SARS was identified, all patients in the ICU were considered to have been potentially exposed. To prevent spread of SARS, we closed the ICU to admissions and discharges and implemented strict respiratory and contact precautions for all remaining patients. We quarantined 69 healthcare workers who were considered to be at high risk for developing SARS. On the basis of our understanding of disease transmission, we arbitrarily decided that persons at high risk included anyone who had entered the index patient’s room or who had been in the ICU for >4 hours during the patient’s 30.75-h stay.

## Methods

After research ethics board approval and informed consent, two researchers used a structured questionnaire to interview quarantined healthcare workers. The questionnaire elicited demographic information, details about health, and information about exposure to the index patient. Time of exposure was categorized as follows: <1 min, 1–10 min, 11–30 min, 31–60 min, 1–4 h, or >4 h. Exposure proximity, procedures performed, and infection-control precautions were documented. Each healthcare worker was asked about symptoms suggestive of SARS that developed during or after the quarantine period.

For healthcare workers in whom suspected or probable SARS developed, additional data were collected about the nature and course of their illness. Suspected and probable SARS were defined according to the definitions issued by the World Health Organization (WHO) (*7*). Symptoms of suspected SARS were a fever >38°C, respiratory symptoms, and an epidemiologic link with a SARS patient; all quarantined healthcare workers were considered to have an epidemiologic link on the basis of contact with the index patient. Probable SARS was defined as suspected SARS with radiographic lung infiltrates.

### Statistics

All data were entered into an Access (Microsoft Corp., Redman, WA) database by using double data entry technique and analyzed by using SAS version 8.0 (SAS Institute, Inc., Cary, NC). For comparisons of characteristics of healthcare workers with SARS to those of healthcare workers without SARS, we used the two-sample t test for normally distributed variables, Wilcoxon rank sum test for ordinal and skewed continuous variables, and Fisher exact test for categorical variables. Two-sided tests were used for all comparisons. A p value of <0.05 was considered to be statistically significant. Classification and regression tree methods were used to identify predictors of developing SARS (*8*). The healthcare workers were divided into two groups by examining all possible cutpoints of all predictor variables to find the cutpoint of a predictor variable that resulted in the largest difference in the probabilities of developing SARS between the two resulting subgroups. This procedure was performed repeatedly for each resulting subgroup until all members of the subgroup had the same SARS status or the subgroup was too small to warrant further splitting.

## Results

Of the 69 quarantined patients, 63 were interviewed. Five declined, and one could not be contacted. SARS did not develop in healthcare workers who were not quarantined and patients who had been in the unit at the time of the exposure.

### SARS Development

SARS developed in 7 of the 69 quarantined healthcare workers (6 probable, 1 suspected; Table 1). One healthcare worker had a history of type II diabetes mellitus; all other healthcare workers were previously healthy. The median time from exposure to the index patient to onset of symptoms was 5 days (range 3–8 days). All probable case-patients were hospitalized and required oxygen but did not require ICU care. Treatment with levofloxacin (500 mg once a day for 7 days) and ribavirin (2,000–2,200 mg loading dose followed by 1,200 mg every 6 h for 4 days and subsequent tapering off) was administered to all admitted case-patients, and all but one received systemic corticosteroids (1 mg/kg prednisone or equivalent once a day for 5 days with subsequent tapering off). The median hospital stay was 19.5 days (range 13–25 days). All case-patients were discharged. However, 28–32 days after discharge, all reported continued dyspnea with exercise.

**Table 1:** Description of healthcare workers in whom severe acute respiratory syndrome developed^a^

Patients	Occupation	Duration of exposure to index patient	Precautions	Special considerations	
Patient 1	Registered nurse	22 h	Gown, gloves, surgical mask^b^	• Present during intubation of airway • Performed all primary nursing activities on 2 shifts	
Patient 2	ICU nurse	31–60 min	N-95 mask, gown, gloves	• Performed difficult intubation of airway	
Patient 3	Registered nurse	None	Not applicable	• Assigned to patient 3 rooms down hall from index patient	
Patient 4	Registered nurse	31–60 min	Gown, gloves, surgical mask	• Assisted primary nurse with bathing of index patient	
Patient 5	Anesthetist	10–30 min	Gown, gloves, surgical mask	• Performed difficult intubation of airway	
Patient 6	Respiratory therapist	4 h	none	• Instituted NPPV • Inserted arterial line	
Patient 7^c^	Respiratory therapist	6 h	Gown, gloves^b^	• Instituted NPPV • Frequently manipulated oxygen mask	

^a^ICU, intensive-care unit; NPPV, noninvasive positive-pressure ventilation. ^b^Denotes precautions that were taken by the healthcare worker sometimes but not always during exposure. ^c^Patient 7 has been classified as a suspected case, as she did not have radiographic lung infiltrates.

### Room Visitation

Thirty-one healthcare workers had entered the index patient’s room; SARS developed in 6 (19%). The contact characteristics and infection control precautions used by the healthcare workers who entered the patient’s room are shown in Table 2. All six healthcare workers in whom SARS developed and who entered the patient’s room reported being present >11 min; three were in the room for >4 hours. SARS attack rates were higher among healthcare workers who spent more time in the index patient’s room; in addition, a dose-response effect occurred between duration of exposure and risk of developing SARS (Table 3).

**Table 2:** Contact characteristics and infection control precautions for 31 healthcare workers who entered index patient’s room^a^

Exposure type	No. healthcare workers with exposure	No. (%) exposed healthcare workers with SARS
Entry into room	31	6 (19)
Contact duration for those entering the room		
<10 min	11	0
11–30 min	8	1 (12.5)
31 min to 4 h	8	2 (25)
>4 h	4	3 (75)
Nature of contact		
Touched patient	19	6 (32)
Contact with mucous membranes	10	4 (40)
Performed procedure involving contact with mucous membranes or respiratory secretions	15	6 (40)
Present during NPPV	22	4 (18)
Performed or assisted intubation	5	3 (60)
Infection control precautions used during exposure		
Always wore at least:		
Gloves	15	3 (20)
Gown and gloves	15	3 (20)
Any mask (N-95 or surgical mask)	13	3 (23)
Gown, gloves, and N-95 mask	6	1 (17)
Gown, gloves, and surgical mask	6	2 (30)
Gown, gloves, and any mask	12	3 (25)
No precautions	8	1 (12.5)

^a^NPPV, noninvasive positive-pressure ventilation.

**Table 3:** Development of severe acute respiratory syndrome (SARS) in healthcare workers, depending on time spent in index patient’s room (N=31)^a^

Time spent in index patient’s room	No. (%) healthcare workers with specified exposure with SARS	No. (%) healthcare workers without specified exposure with SARS	Odds of developing SARS after specified exposure	95% CI for OR	p value	
<10 min	0/11	6/20 (30)	0.097^b^	(0.005 to 1.91)^b^	0.047	
>31 min	5/12 (42)	1/19 (5)	12.9	(1.27 to 131)	0.014	
>4 h	3/4 (75)	3/27 (11)	24.0	(1.85 to 311)	0.003	

^a^CI, confidence interval; OR, odds ratio. ^b^These logit estimators use a correction of 0.5 in every cell of the table that contains a zero.

### Contact with Index Patient

All six healthcare workers with SARS who entered the index patient’s room also touched the patient, and all reported performing a procedure that involved contact with the patient’s mucous membranes or respiratory secretions (Table 2). Three of the six healthcare workers reported wearing gloves during this contact. In contrast, of the 13 healthcare workers without SARS, 12 (92%) used gloves when touching the patient (odds ratio [OR] 0.08, 95% confidence interval [CI] 0.01 to 1.11, p=0.04). Selected contact characteristics predictive of the development of SARS in healthcare workers who entered the patient’s room appear in the Figure.

**Figure:**
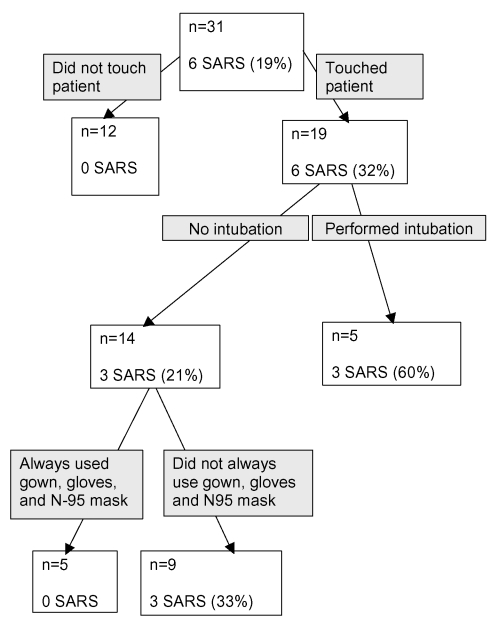
Regression tree describing selected contact characteristics in healthcare workers who entered the index patient’s room. Does not include results for one healthcare worker who had no history of entering the index patient’s room but nevertheless acquired severe acute respiratory syndrome.

SARS developed in three of the five persons present during the endotracheal intubation of the patient. During this procedure, the patient’s respiratory secretions were splashed onto the uncovered cheek of one of the healthcare workers. No other healthcare worker reported direct skin exposure to the patient’s bodily secretions at any time during his admission. Two of the three persons in whom SARS developed after the endotracheal intubation wore a gown, surgical mask, and gloves; one healthcare worker wore a gown, gloves, and N-95 mask. Of the two healthcare workers present during endotracheal intubation in whom SARS did not develop, one was a postgraduate medical trainee who assisted with manual ventilation (bag-valve-mask ventilation using a Laerdal bag) and was positioned to the side of the patient rather than directly over the patient’s head. This healthcare worker wore gown, gloves, and surgical mask during the procedure. The second worker was a respiratory therapist who helped prepare the necessary equipment while wearing gown, gloves, and an N-95 mask.

Of the healthcare workers who entered the index patient’s room, 22 were present at some time during the administration of noninvasive positive-pressure ventilation (NPPV), and SARS developed in 4 (18%). Each of these 4 healthcare workers, but only 1 of the 18 healthcare workers who remained well, reported being present in the room for >31 minutes during the administration of NPPV (OR 105, 95% CI 3 to 3,035, p <0.0001). The one worker in whom SARS did not develop despite being present during NPPV therapy for >31 minutes wore a surgical mask, gown, and gloves. One of the 4 healthcare workers in whom SARS developed and 4 of the 18 healthcare workers who remained well wore an N-95 mask during NPPV administration.

### No Room Visitation

SARS developed in one quarantined healthcare worker (a nurse) who had not entered the index patient’s room; the disease did not occur in any other healthcare workers who had not touched or had close contact with the index patient. The nurse was present in the ICU for 18.75 h (two shifts) during the patient’s admission. Of note, after the endotracheal intubation of the index patient, the physician who performed this procedure entered the room where the nurse was caring for another patient. Neither the nurse nor the physician recalled direct contact, and they were certain that the physician had changed gloves and gown before room entry. This nurse had no other epidemiologic risk to explain the development of SARS.

### Other Observations

One healthcare worker spent >4 hours with the index patient; however, SARS did not develop in this worker. This worker wore an N-95 mask, gloves, and gown during exposure and was not present during the endotracheal intubation or during the administration of NPPV. SARS did develop in another healthcare worker who performed the endotracheal intubation while wearing an N-95 mask, gown, and gloves.

## Discussion

Our results suggest that proximity and duration of contact to a patient with SARS are associated with risk for viral transmission, an observation suggested by others (*2*–*4*). In addition, certain procedures, such as endotracheal intubation, pose increased risk. These findings may be predictable given that SARS is thought to spread primarily by large droplets (*9*).

Three of the six persons in whom SARS developed after entering the index patient’s room may not have adhered to standard MRSA precautions in that they performed procedures which involved contact with mucous membranes without wearing gloves. Furthermore, we were unable to determine if hand washing impacted SARS transmission, as this information was not collected.

During our study, we made two important observations. First, SARS developed in one healthcare worker despite the fact that the worker wore an N-95 mask, gown, and gloves. Second, SARS developed in another healthcare worker who had no identified contact with the index patient or with any other persons known to have SARS. In the case of the first healthcare worker, the absence of eye protection may have contributed to disease transmission. In addition, although this person wore an N-95 mask while in the patient’s room, he had not been fit-tested for this mask; however, fit-testing should not be necessary if the SARS-associated coronavirus is spread by large droplets (*6*). As a result of this and similar episodes of SARS transmission in the Toronto area (*10*), the province of Ontario has now made specific recommendations for healthcare workers performing intubation that involve increased protection (available from: URL: www.sars.medtau.org) (*11*), and protective eye wear is currently mandated for patient encounters. In the second case, transmission could have occurred in a number of possible routes. The nurse may have come within sufficient range of the SARS patient to be exposed to large droplets. Recent reports indicate that the virus may survive for several hours on fomites or in body secretions (*12*) and raise the possibility of transmission by indirect contact with contaminated objects or of inadvertent carriage and spread by another healthcare worker. Fecal transmission is unlikely as the patient did not have a bowel movement during his stay. True airborne spread may also have occurred. Although evidence does not support this route of transmission for the SARS-associated coronavirus, existing literature suggests that other coronaviruses may be spread by an airborne route in certain circumstances (*13*).

Given our lack of knowledge about the transmissibility of SARS at the time this exposure occurred, we made a conservative decision to quarantine for 10 days all persons who were in the unit for at least 4 h or who had a history of entry into the affected patient’s room. In addition, we closed the ICU to admissions and discharges for a 10-day period, markedly affecting our institution’s ability to deliver health care. In fact, during the Toronto outbreak, several of the city’s ICUs were closed as a result of quarantine and illness in staff with similar consequences (*14*); by infecting healthcare workers, SARS has an impact on the health of an entire community. A less aggressive quarantine approach may have been as effective in controlling transmission and allowed more staff to be available for work. For instance, only persons who have had direct contact with the patient (i.e., entered the patient’s room) could have been quarantined. If we had taken this approach, the quarantine would have excluded six persons with SARS from the workplace but only removed 25 of the 62 persons who remained well. However, this approach would have missed one healthcare worker in whom SARS developed. Another approach might be to monitor staff closely for SARS-related symptoms while they continue their usual activities and quarantine only those in whom symptoms occur. This approach would require evidence that SARS cannot be transmitted before symptom onset, confidence in the facility’s ability to identify symptomatic staff, and reliability of healthcare workers in reporting symptoms. We think that our quarantine approach prevented secondary spread of illness to other persons who may have come in contact with the workers in whom SARS developed.

Our study involved a small number of cases, and definitive conclusions cannot be drawn from a report of this size. For example, although SARS developed in our staff within the 10-day quarantine period, others have demonstrated that the time period from infection to onset of symptoms may be >10 days (*15*). One of the strengths of our study is that the exposure occurred during a defined period in a contained unit, and as such, there is less potential for confounding caused by the exposure of healthcare workers to multiple SARS patients.

Our observations emphasize the consequences of missing the diagnosis of SARS for even a relatively brief period. In our experience, we would make the following recommendations. First, the possibility of unexpected exposure of healthcare workers to patients with SARS should be anticipated, and once such exposure is recognized, those deemed to be at risk for SARS transmission should be promptly quarantined. Second, vigilant surveillance for symptoms of SARS must be maintained by all healthcare workers who work in institutions with SARS patients; SARS may develop in healthcare workers even when they do not have direct exposure to patients with SARS. In addition, protocols for managing patients with SARS should include not only contact and respiratory precautions but also procedures that minimize patient contact since duration and proximity of contact increase the risk for transmission of SARS. Finally, additional precautions should be taken when performing high-risk procedures, such as endotracheal intubation (*11*).

Though many of the healthcare workers in our ICU were exposed to the patient with SARS, our experience suggests that the greatest risk for SARS transmission occurs in those healthcare workers with prolonged exposure or direct physical contact with the patient. Use of gowns, gloves, and masks as barriers appears to reduce the risk for SARS transmission in most but not all situations. Additional information will be needed to determine if modes of transmission beyond droplet spread are important. We think this information will be helpful to institutions dealing with similar exposures to patients with SARS and developing quarantine protocols.
